# Transition between preclinical and clinical training: Perception of dental students regarding the adoption of ergonomic principles

**DOI:** 10.1371/journal.pone.0282718

**Published:** 2023-03-09

**Authors:** Júlia Carrer Hallak, Franciele de Souza Ferreira, Caroline Anselmi de Oliveira, Júlia Margato Pazos, Tamíris da Costa Neves, Patrícia Petromilli Nordi Sasso Garcia

**Affiliations:** 1 Department of Social Dentistry, São Paulo State University (Unesp), School of Dentistry, Araraquara, SP, Brazil; 2 Department of Morphology and Children’s Clinic, São Paulo State University (Unesp), School of Dentistry, Araraquara, SP, Brazil; AIIMS: All India Institute of Medical Sciences, INDIA

## Abstract

**Objective:**

This study aimed to evaluate the perceptions of third-year dental students regarding the application of ergonomic principles in the transition between preclinical and clinical training in Restorative Dentistry.

**Methods:**

We conducted a qualitative observational cross-sectional study. The sample consisted of forty-six third-year dental students at São Paulo State University (Unesp), School of Dentistry, Araraquara. Data was collected using an individual interview recorded on a digital voice recorder. A script containing questions related to the process of adaptation of students to clinical care with a view to ergonomic work posture was used. Data analysis was based on the quali-quantitative technique of Discourse of the Collective Subject (DCS), using Qualiquantisoft®.

**Results:**

Most students (97.80%) perceived the need for an adaptation period in the transition from the preclinic to the clinic regarding ergonomic posture requirements; a part of them (45.65%) claimed that they still could not adapt, primarily due to the difference between the laboratory and clinic in the workstation (50.00%). Some students suggested longer preclinical training in a clinical environment to facilitate this transition (21.74%). The dental stool (32.60%) and the dental chair (21.74%) were the external factors that contributed most to making this transition difficult. The difficulty of the restorative dentistry procedure (10.87%) also interfered with posture. Additionally, the most challenging ergonomic posture requirements in the transition period were maintaining 30 to 40 cm between the patient’s mouth and operator’s eyes (45.65%), positioning the patient in the dental chair correctly (15.22%), and working with the elbows close to the body (15.22%).

**Conclusion:**

Most students perceived the need for an adaptation period in the preclinical transition to the clinic, attributing difficulties to adopt the ergonomic posture requirements, to use the workstation and to perform the procedures on real patients.

## Introduction

Dental education aims at the professional’s technical-scientific, humanistic, and social training [1]. Therefore, in addition to theoretical knowledge, it is essential to develop motor skills, behavior, and professional values [2].

It is recommended that students incorporate appropriate postural habits as they acquire their professional skills [3]. This is done in some Dental Schools where manual skills’ training happens simultaneously with the teaching of Ergonomics [4, 5], science that studies the adaptation of work to the worker [6].

Dental Ergonomics studies the dentist and his team [7] to reduce the cognitive and physical stress, helping to prevent occupational diseases [8] and improving the productivity and quality of dental procedures [8, 9].

Araraquara School of Dentistry (São Paulo State University/UNESP) offers the Ergonomics in Dentistry course for the students in the second year of graduation. Various ergonomics topics are taught, including working position and requirements for ergonomic posture in dentistry [9]. These 10 requirements proposed by Porto (1994) [6] are: 1) Sitting with things parallel to the ground, forming an angle of 90° with the legs. 2) Sit down slightly straight, leaning on the backrest of the dental stool, in the renal region and with the head forward and down. 3) Elbows close to the body. 4) The patient´s mouth should be at the height of the dentist´s knees. 5) The patient´s head height should allow one of the operator legs to be under the patient chair seat without pressure. 6) Patient head positioned down for upper jaw work and patient head positioned up for lower jaw work. 7) Reflector in front of the patient´s mouth for work on the maxilla and reflector perpendicular to the patient´s head for work on the mandible. 8) Maintaining 30 to 40 cm between the operator’s eyes and the patient’s mouth. 9) Instruments and materials must be positioned to be reached with a maximum of forearm movement. 10) Use of high-power suction.

This Ergonomic in Dentistry course has 15 hours of theoretical classes and 90 hours of practical activities taught simultaneously with preclinical training in Restorative Dentistry I course. In this preclinical training students begin to develop their psychomotor skills [10–14] and to face the first challenges in performing dental procedures [11]. This moment is important to the development and acquisition of motor and visual skills, hand-eye coordination, spatial consciousness, visualization of three-dimensional oral structures in fine detail [2, 4, 12] and to prepare students for patient care in the medium-term.

The development of psychomotor skills happens in a safer way [10] during the preclinical training because the students work in dental mannequins. However, when clinical training begins students perform dental treatment on real patients, where an error could harm their oral or general health [10, 13, 14]. Therefore, the transition period between preclinical and clinical training can be extremely stressful for students.

Thus, observing students’ perceptions regarding the application of ergonomic posture requirements in the transition between preclinical and clinical Restorative Dentistry is important, considering that the difficulty faced in the transition process is influenced by acquired preclinical skills [14] and that adequate preclinical training can help in a smooth transition between the preclinical and clinical stages [10]. This information can be valuable for critical thinking in preclinical ergonomics teaching and implementing innovations [13]. It is worth emphasizing that no studies specifically focused on this transition phase were found in the literature.

This study aimed to observe the perceptions of third-year dental students about the application of ergonomic principles during the transition phase between preclinical and clinical training in Restorative Dentistry.

## Materials & methods

### Sample and study design

This was a cross-sectional observational study, qualitative in nature, with a non-probabilistic sample design. The sample consisted of third-year undergraduate dental students of both sexes from the São Paulo State University (UNESP), School of Dentistry, Araraquara (N = 46) with ages ranging from 19 to 25 years, and the majority (80.00%) being women compared to men (20.00%). This sample was chosen because these students went through the transition phase from preclinical to clinical training in the Restorative Dentistry II course. This course is annual and is taught in the first and second semester of the third year of the dental graduation course at the School of Dentistry of Araraquara. It is worth emphasizing that, as in the preclinical, in this transition phase, the students also carry out practical ergonomics training simultaneously with the clinical activities of the Restorative Dentistry course.

The research was conducted in the first week of the second semester, so the students would have already interacted with clinical activities and developed their perception of the transition phase.

This study was approved by the Research Ethics Committee of the São Paulo State University (UNESP), School of Dentistry, Araraquara (CAAE Registry No. 90949018.6.0000.5416). A written informed consent was obtained from participants of this study.

### Data collection

Data were collected through personal and individual interview recorded on a digital recorder. We chose this method because it ensures face-to-face interaction, allowing students to express spontaneously their thoughts and arguments in detail and free of any interference [15].

The interviews were conducted in Portuguese, the native language of Brazil. During the interview, a script was used containing questions related to the students’ adaptation process to clinical care from the point of view of the ergonomic work posture.

After the interviews, the recordings were transferred from the recorder to the computer to transcribe the speeches for further analysis.

### Questions asked in the interview

The questions were formulated in an open-ended and objective way, as recommended by Lefevre Lefevre (2012) [15]. After been elaborated, the questions were pre-tested in a pilot study.

1) Did you need time to adapt to the transition from preclinical restorative dentistry to the restorative dentistry clinic, considering the principles of ergonomics? If so, how long? 2) Based on question 1, why did you need time to adjust? 3) Considering the transition from preclinical restorative dentistry to the restorative dentistry clinic, what do you feel was missing concerning ergonomics learning that could have facilitated this process? 4) Among the 10 requirements for obtaining an ergonomic posture, which has been the most challenging for you to adopt while attending the Restorative Dentistry clinic? Why? 5) Do you think the factors related to dental equipment (dental chair, dental stool, dental unit, reflector, auxiliary table, and auxiliary unit) have compromised your adoption of learned ergonomic posture requirements? If so, cite the main factor(s) and the reason for your choice. 6) Do you think performing clinical procedures on real patients influences the adoption of ergonomic posture requirements? If so, in what manner?

### Data analysis

Data analysis was based on the qualitative and quantitative technique of the Discourse of the Collective Subject (DCS), and was carried out with the Qualiquantisoft®, which is a computer program that was developed to facilitate the analysis of data from qualitative research that use the DCS technique [15].

This technique is based on social representation theory, which is a socially shared idea. The raw data obtained from each individual statement go through a process that results in collective discourse. Each of these collective discourses makes possible to represent a certain opinion or position of the group of people evaluated, based on the literal response of the most significant content of the discourse [16].

In addition to the qualitative data analysis, a quantitative analysis was carried out using descriptive statistics in order to obtain a relative frequency distribution of the results organized by the categories of each question.

## Results

The response rate was 61.33% (46 students). Table 1 presents the quantitative results.

**Table 1:** Summary of the Discourse of the Collective Subject (DCS) questionnaire results organized by the categories associated with each question.

Questions	%
**1A) Did you need time to adapt to the transition from preclinical restorative dentistry to the restorative dentistry clinic, considering the principles of ergonomics?**	
A. Yes.	97.80
B. No.	2.20
**1B) If so, how long?**	
A. Two weeks.	6.52
B. Three weeks.	6.52
C. One month.	13.04
D. Two months.	15.22
E. One semester.	4.35
F. I still haven’t been able to adapt.	45.65
G. Did not answer the question.	8.70
**2) Based on question 1, why did you need time to adjust?**	
A. Difference between the workplace in the laboratory and the clinic.	50.00
B. Work with an assistant.	4.34
C. Existence of concern about the procedure being performed in the patient’s mouth at the clinic.	17.39
D. Difference in teaching methodology.	6.52
E. Difficulty seeing.	4.35
F. Did not answer the question.	17.39
**3) Considering the transition from preclinical restorative dentistry to the restorative dentistry clinic, what do you feel was missing in terms of ergonomics learning that could have facilitated this process?**	
A. I don’t feel that any teaching was missing; it was enough.	71.74
B. More practical classes in Ergonomics in Dentistry at the clinic.	21.74
C. If teaching had been more personal.	2.17
D. Standardization of Ergonomics Teaching in Dentistry I and II.	2.17
E. Did not answer the question.	2.17
**4) Among the 10 requirements for obtaining an ergonomic posture, which has been the most challenging for you to adopt while attending the Restorative Dentistry clinic?**	
A. Maintaining 30 to 40 cm between the operator’s eyes and the patient’s mouth.	45.65
B. Working seated with the back against the back of the dental stool.	13.04
C. Position the patient properly in the dental chair.	15.22
D. Working with the elbows close to the body	15.22
E. Sit with thighs parallel to the floor, forming a 90° angle with the legs.	2.17
F. Did not answer the question.	8.70
**5a) Do you think the factors related to dental equipment (dental chair, dental stool, dental unit, reflector, auxiliary table, and auxiliary unit) have compromised your adoption of learned ergonomic posture requirements?**	
A. Yes.	73.91
B. No.	23.91
C. Did not answer the question.	2.17
**5b) If so, cite the main factor and the reason for your choice.**	
A. Dental Unit.	8.70
B. Dental stool.	32.61
C. Auxiliary table.	8.70
D. Dental chair.	21.74
E. The factors didn’t compromise.	19.57
F. Did not answer the question.	8.70
**6a) Do you think performing clinical procedures on real patients influences the adoption of ergonomic posture requirements?**	
A. Yes.	91.30
B. No.	2.17
C. Did not answer the question.	6.52
**6b) If so, in what manner?**	
A. The difference between the dental mannequin and the patient.	69.57
B. The concentration on the procedure being performed.	10.87
C. Difficulty in vision and access.	10.87
D. Did not answer the question.	8.70

It was verified that the vast majority of students (97.80%) needed some time to adapt in the transition between the preclinical to the clinic. Almost half of the evaluated students (45.65%) had not adapted yet to the preclinical transition during the interview. One student said *“I think I have not fully adapted either”*, and another student stated *“Well*, *I think am still adapting to this day*, *to be very honest*.*”*. Of those who had already adapted, the most reported period for this adaptation was 2 months (15.22%) followed by 1 month (13.04%).

The main reason (50.00%) for the need to adapt in the preclinical and clinical transition phase was the difference between the workstation of the preclinical laboratory and of the clinic. Some students reported: *“Because the dental chair is very different from the mannequin*, *with the mannequin is just the head*, *but the chair has the whole body”*, *“Because*, *mainly with the change of equipment*, *I found it more difficult*, *especially to position myself in 9 o’clock*…*”*, *“Because the equipment is very wide*, *the leg stays very close under the chair*, *then I thought it more difficult*.*”*, *“I think it is a little different the way you organize yourself in the lab and the clinic; the principles are the same*, *but the things’ order is a little different*.*”*.

This reason was followed by the students’ concern about the procedure they were doing in the patient’s mouth (17.39%) *“*…*when you are in the clinic you have other*, *let’s say*, *worries*, *then you end up forgetting a little of the ergonomic postures requirements*…*”*, *“*…*if there is a tooth that I have more difficulty treating*, *I care more about doing it than paying attention to my posture*.*”*, *“*…*it is a huge responsibility because you’re dealing with someone else’s life*…*”*, *“Because during the clinic there is the pressure of the patient being there*.*”*.

Most of the students (71.74%) reported that the knowledge acquired in Ergonomics in Dentistry course was sufficient for the preclinical and clinical transition *“The teaching itself*, *I believe*, *was all good; it is more about the concern of practice with the patient*.*”*, *“No*! *I think the position in the laboratory itself is very different from the clinic*. *But I think nothing was missing*.*”*. Despite this, some students (21.74%) suggested increasing the number of ergonomics classes *“I think I should have taken more ergonomics classes at the clinic*. *I think in the lab*, *it’s hard because it’s nothing like a dental unit*, *mainly because you have to lift and lower the dental chair*, *the backrest*.*”*, *“Because I think there was few classes in the clinic in terms of ergonomics*, *I needed more*.*”*, *“I think everything should have been taught with the mannequin in the dental chair*, *in the clinic”*, *“I think we could have gone more often to the clinic than stayed just in the laboratory*.*”*

The requirement most cited (45.65%) as difficult to apply was the maintenance the distance of 30 to 40 cm between the operator’s eyes and the patient’s mouth *“Because I feel that*, *regardless of the way I adjust the chair and the patient’s position*, *I always need to come closer to the patient to do a good job*,*”*, *“I think a little difficult to keep the distance of 40 cm*. *When I realize*, *I am already that close of the patient’s mouth”*, *“it seems that it gives more confidence to* see *closely*, *to get closer to what you are doing”*, *“Because the area we work in is small*, *sometimes you end up wanting to see more details*, *but when it is too far away*, *it is harder*.*”*, *“I think more about my insecurity*.*”*. This requirement was followed by the requirements *“*position the patient properly in the dental chair*”* (15.22%) and *“working with the elbows close to the body”* (15.22%).

Most students (73.91%) realized that the factors related to dental equipment in the clinic compromised the adoption of their ergonomic posture. We observe that the dental stool (32.61%) and the dental chair (21.74%) were the most cited. About the dental stool, some students related: *“The dental stool is horrible*. *It is the main problem*.*”*, *“The dental stool is the one that influences the most*, *because there are several people who use the same dental stool; sometimes I realize that we arrive and already pack things and forget to adjust the backrest in a way that back is well supported*, *and it ends up getting more back or forward than needed*, *and then you end up sitting wrong*.*”*. About the dental chair, some students reported: *“The seat of dental stool is too big*, *making it difficult for the team to fit in*. *In addition*, *the patient chair is also very large”*, *“The backrest of the dental chair does not let my leg to get down there*, *and I’m small*, *imagine if I was tall*?*”*.

The majority of the students (91.30%) reported that the difficulty in the procedure performed interfered negatively with their posture, were most students (69.57%) attributed this difficulty to the difference between the preclinical mannequin and the patient in the clinic. *“The difference of mannequin and dental chair*, *and the position that we have to stay is also a little different*.*”*, *“I could throw the mannequin upside down*, *and it would not say anything*, *but the patient can complain*.*”*, *“It is different because you cannot position yourself anyway*, *like in the mannequin*, *because it is a real patient now; it is much easier for you to manipulate a mannequin than you put the patient in a chair in a certain position*, *normally the patient does not want to be in that position*.*”*

## Discussion

The transition period between the preclinical and clinical phases is crucial since students are exposed to factors that can hinder or facilitate the adaptation process, such as applying theoretical concepts to practice. So, the present study aimed to qualitatively observe, through the Discourse of the Collective Subject (DCS), the perceptions of third-year undergraduate dental students on applying ergonomic principles during the transitional phase between preclinical and clinical training in Restorative Dentistry.

The Discourse of the Collective Subject (DCS) is a qualitative data analysis method that uses collective thinking to explore the social work field and rescue the differences and similarities between the views of the participating subjects [15]. This analysis has been indicated when it is necessary to deepen the understanding of the behaviors of a specific group and was used in previous researches to evaluate dental students [17–19].

From the data obtained, we verified that many students (45.65%) had not fully adapt from preclinical to clinical training. In our school the clinical training begins in the first semester of the third year. However, our data were not collected in this initial period to give some adaptation time to students, once they were having their first contact with the dental care in clinics. It is important to emphasize that even after one academic semester, the students had not completely adapted to clinical care.

We found that the main reason cited by students for this difficulty of adaptation was the difference between the workstation of the preclinical and clinical training. This because the workstation in the laboratory is limited to dental stool, lighting and fixed bench, and in the clinic there are dental chair, dental stool, lighting, dental unit, auxiliary table and other elements. Another reason for this difficulty of adaptation was the students’ concern about the procedure they were doing in the patient’s mouth. The fact that procedures become irreversible when caring for real patients can cause stress in students [10, 13, 14].

We asked the students what was missing from the Ergonomics in Dentistry taught that could have helped the transition from preclinic to the clinic. More than half of the students (71.74%) reported that the knowledge acquired in Ergonomics in Dentistry was sufficient for the preclinical and clinical transition. Some students (21.74%) suggested increasing the number of preclinical classes in the clinical environment rather than the laboratory. The implementation of preclinical practical classes in the clinic workstation would be beneficial. The students would have the ergonomic knowledge applied in the clinical workstation before starting the clinical activities themselves, being able to improve their compliance to the principles of ergonomics and work in a healthier way.

The ergonomic posture requirement considered as the most challenging to implement in the clinical environment was the maintenance of the distance of 30 to 40 cm between the patient’s mouth and the operator’s eyes. The need for to stay closer to the operative field could be related to the difficulty of visualizing to perform the procedure. This difficulty in visualizing the operative field was reported by some students when asked about how performing clinical procedures on real patient could interfere in the adoption of an ergonomic posture (question 6b).

Kamal et al. (2020) [18] observed that the implementation of magnification loupes in pre-clinical training period could be beneficial. Pazos et al. (2020) [19] found that using both the Galilean and Keplerian magnification systems improved the working posture during the performance of simulated clinical procedures. Thereby, the implementation of magnification loupes for better visualization of the operative field may be positive to help the students in maintaining the distance of 30 to 40cm between the patient´s mouth and the operator´s eyes and, consequently, improve the adoption of ergonomic posture [20, 21].

Students were asked about the factors related to dental equipment and its relationship with working posture. Characteristics of the dental equipment can interfere with the adoption of ergonomic posture. The dental chair and the dental stool must allow adequate adjustment to guarantee the correct positioning of the patient and the student, respectively. The dental unit and the auxiliary table must be properly positioned to prevent a great lateral inclination of the student’s spine. The lighting must have good quality and mobility to adequately illuminate the operative field and facilitate visualization of the tooth to be treated.

The items cited by students as being challenging during the transition phase in relation to the adoption of ergonomic posture were dental stool (32.61%), dental chair (21.74%), dental unit (8.70%) and auxiliary table (8.70%). We observe that the dental stool is one of the items of the dental equipment most reported by students as being challenging during the transition phase. On the other hand, the lighting was not mentioned and this fact may be related to the students’ lack of perception of the influence of this item on the visualization of the work field and, consequently, on their posture.

In our school, we use the conventional dental stool, both in the pre-clinical laboratory and in the clinical. However, the manufacturer of the dental stool used in the laboratory is different from the one used in the clinic and this can have caused difficulties for the students to adopt ergonomic posture.

According to Gouvêa et al. [22] the saddle stools are more favorable to dental students when compared to conventional ones and may be beneficial for students´ compliance to ergonomic posture requirements in the clinic. This stool offers a comfortable posture as it bends the pelvis into an almost neutral position, simulating a standing position with well supported legs and thighs. This position takes the natural curvature of the spine and keeps the shoulder-neck area upright [23], which is the most suitable posture for the lumbopelvic and cervicothoracic region [24].

When questioned about the influence of performing clinical procedures on real patients on the adoption of ergonomic posture requirements most students (91.30%) reported that the difficulty in the procedure performed interfered negatively with their posture, and many students (69.57%) attributed this difficulty to the difference between the dental mannequin and the real patient.

Presoto et al. [11] and Garcia et al. [9] also noted that the degree of complexity related to the procedures performed may interfere with adopting an ergonomic posture. Thus, professors in clinical training must identify which stage and procedure students have greater difficulty in, so that they can help them to overcome them [10].

To the best of our knowledge there are no other studies that have qualitatively evaluated the students’ perceptions of difficulties during the transition from preclinical to clinical training regarding the adoption of ergonomic posture requirements. Thus, the comparison of our findings with previous studies was not possible.

The limitation of this study is related to the non-probabilistic sampling design, where just students from School of Dentistry of Araraquara were included. It is possible that individuals from schools with different philosophies perceive other difficulties when applying ergonomic posture requirements. However, considering the scarcity of works on this subject, this study makes an important contribution to the area.

The results obtained in this research allows professors from different institutions to plan strategies to facilitate the adoption of ergonomic posture requirements, circumventing the difficulties observed in the transition period between the pre-clinical and clinical phases. Thus, we believe that ergonomic principles would be more well-established in dental students’ routine, allowing them to start their professional life more aware of their occupational health, prolonging the exercise of their profession.

## Conclusions

Students perceived the need for a period of adaptation during the preclinical transition concerning the ergonomic posture requirements, mainly due to the difference in the workstation between the laboratory and the clinic. Furthermore, they suggested a more extended period of preclinical training in the clinical environment to facilitate this transition. Additionally, they pointed out that the dental stool and the dental chair were the external factors that most hindered the transition period and that the difficulty of the Restorative Dentistry procedure also interfered with adopting an ergonomic posture. Lastly, they considered the maintenance of the 30 to 40 cm between the mouth of the patient and the eyes of the operator, and the back supported on the backrest of the dental stool as the most challenging ergonomic posture requirements in the transition period.

## Supporting information

S1 Raw data (XLSX)
